# Informal relationship patterns among staff of local health and non-health organizations in Thailand

**DOI:** 10.1186/s12913-015-0781-8

**Published:** 2015-03-20

**Authors:** Mano Maneechay, Krit Pongpirul

**Affiliations:** Department of Preventive and Social Medicine, Faculty of Medicine, Chulalongkorn University, 1873 Rama IV Rd., Or Por Ror Building, 19th Floor, Pathumwan, Bangkok, 10330 Thailand; Khok Samrong District Public Health Office, Lopburi, Thailand; Department of International Health, Johns Hopkins Bloomberg School of Public Health, Baltimore, MD USA; Bumrungrad International Hospital, Bangkok, Thailand; Thailand Research Center for Health Services System (TRC-HS), Faculty of Medicine, Chulalongkorn University, Bangkok, Thailand

**Keywords:** Informal relationship, Local health services system, Health decentralization

## Abstract

**Background:**

Co-operation among staff of local government agencies is essential for good local health services system, especially in small communities. This study aims to explore possible informal relationship patterns among staff of local health and non-health organizations in the context of health decentralization in Thailand.

**Methods:**

Tambon Health Promoting Hospital (THPH) and Sub-district Administrative Organization (SAO) represented local health and non-health organizations, respectively. Based on the finding from qualitative interview of stakeholders, a questionnaire was developed to explore individual and organizational characteristics and informal relationships between staff of both organizations. Respondents were asked to draw ‘relationship lines’ between each staff position of health and non-health organizations. ‘Degree of relationship’ was assessed from the number that respondent assigned to each of the lines (1, friend; 2, second-degree relative; 3, first-degree relative; 4, spouse). The questionnaire was distributed to 748 staff of local health and non-health organizations in 378 Tambons. A panel of seven experts was asked to look at all responded questionnaires to familiarize with the content then discussed about possible categorization of the patterns.

**Results:**

Responses were received from 73.0% (276/378) Tambons and 59.0% (441/748) staff. The informal relationships were classified into four levels: strong, moderate, weak and no informal relationship, mainly because of potential impact on local health services system. Strong informal relationship existed when the Chief Executive of SAO had any relationship degree with any THPH staff. When the Deputy Chief Executive of SAO or Chairman of the SAO Council had such relationship, the Tambon was classified as moderate level. Tambon with some other relationship patterns was categorized as weak. Approximately 58.5, 12.0, 7.4 and 22.2% of the surveyed Tambon have strong, moderate, weak, and no informal relationship, respectively.

**Conclusion:**

The finding suggested that informal relationships between the staff of local health and non-health agencies can potentially affect the operations and development of the local health services system.

**Electronic supplementary material:**

The online version of this article (doi:10.1186/s12913-015-0781-8) contains supplementary material, which is available to authorized users.

## Background

The Thai population has seen a significant improvement in health outcomes mainly as a result of public investment in infrastructure and human resources during the earlier years along with successful introduction of the Universal Coverage (UC) scheme [1]. As central governments depend on local governments to fulfill national policy aims, the other important national agenda was the decentralization of public services to local governments which has been in the Thai constitution since 1997. A broad range of services in social, education, and health areas directed at increasing individuals’ well-being were included [2]. Its aim was to increase public participation in decision making, improve service delivery by fostering greater bottom up accountability [1].

Tambon (sub-district) was considered the most optimal decentralized administrative unit in Thailand. It was governed by Sub-district Administrative Organizations (SAO) under the supervision of Ministry of Interior. This locally elected government body was anticipated to have almost full responsibility of sub-district economic, social, education, and health services. As basic health services have been provided by Tambon Health Promoting Hospital (THPH) under the umbrella of Ministry of Public Health, the initial decentralization strategy was to transfer THPH to be under SAO. Unfortunately, only less than one percent of the Tambons have successfully decentralized with no significant progress over the past five years.

Evidence from the successfully decentralized areas suggested that one of the key factors was having a good relationship between the two organizations [3]. This was not surprising as “organizations are located separately, governed by different laws at various levels of government, and have different intake procedures with incompatible eligibility standards, funding limitations, incompatible professional world views, and so forth” [4]. Co-operation among staff of government agencies is essential for good local health services, especially in small communities. As all organizations have either trivial or vital relationships with other organizations, improving inter-organizational relations has been a common concern of researchers and practitioners for many decades [2]. In significant degree the health services provided by local health organizations relied on the work and resources of non-health organizations [2]. Goals and strategies of individual organizations are likely to explain only a minimal portion of the variance in outcomes [5], which, at worse, could be distorted by ambiguous and contradictory goals of these organizations [6]. Clients are also affected by inter-organizational relations because they are often served by several associated organizations [7]. The increased interest in public service integration has changed the focus from only internal issues to organizational ‘foreign affairs’ [8].

In corporate settings, inter-organizational relations seem to be more clearly ordered than they are in health services [2]. Most leadership theories treat interagency relations as a rational instrument for fulfilling goals, especially resource procurement. Empirical studies have revealed that health service executives devote rather little time to managing the external environment [9].

Links between organizations vary a great deal with regard to the degree of formalization, intensity, reciprocity, standardization, mandatory, and tangibility [7]. In the business sector, much of the real work of a company actually happens because of informal systems [10]. “If the formal organization is the skeleton of a company, the informal is the central nervous system driving the collective thought processes, actions, and reactions of its business units” [10]. Managers therefore try to map social links in order to harness the real power in their company [10].

As health service executives are constantly involved in activities that cross the formal boundaries of the organization [2], this concept could be applied in health service provision by a network of local health and non-health organizations. As supporting evidence in local health service systems has been lacking, this study was aimed to explore possible patterns of informal relationships among staff of local health and non-health organizations in the context of health decentralization in Thailand.

## Methods

This study is part of the Health Systems Management Models at Tambon Level project, financially supported by Health Systems Research Institute (HSRI), Thailand. The first phase of this project was an exploratory situational assessment of local health systems using extensive document analysis and in-depth interview with 23 stakeholders, representing policy, management, and practice viewpoints of health and non-health sectors.

In this second phase, a national survey using a questionnaire based on the qualitative findings (see Additional file 1) was conducted to better understand individual and organizational characteristics of local organizations at Tambon level. Informal relationship between local organizations was one of the key themes that emerged as a critical component of successful decentralization. To deal with such an ill-defined concept, a focus group discussion among investigators with additional review of potential methodologies was conducted and the most optimal approach was chosen. In this part of the questionnaire, respondents were asked to draw all possible ‘relationship lines’ between each pair of SAO and THPH staff positions, along with a number on each line that reflect ‘degree of relationship’ (1, friend; 2, second-degree relative; 3, first-degree relative; 4, spouse).

Multistage cluster sampling was used in the national survey. Twelve out of 18 regions were randomly selected, of which two provinces were randomly chosen. One district was randomly selected from each province. All SAO and THPH in each district were included in this study. With 5% type I error, 5% precision, and 50% prevalence of positive outcome, adjusted with design effect of 1–2 for cluster sampling, the sample size was calculated to be 384–768.

To maximize response rate and quality, the questionnaires were sent to a staff member in each district health office who acted as a local coordinator, responsible for distributing and collecting the questionnaire from SAO and THPH in the area.

While descriptive statistics were used to analyze the questionnaire in the main project, the informal relationship section was separately investigated and presented in this paper. A focus group discussion was conducted among seven (four health and three non-health) local experts who have worked in Tambon for more than 10 years on average. They were asked to look at all returned questionnaires to familiarize themselves with the content. They were then asked to discuss possible categorizations of patterns. The discussion continued until a final conclusion was reached.

### Ethical consideration

This study was approved by the Institutional Review Board of Faculty of Medicine, Chulalongkorn University (IRB No.205/2555). All participants provided written informed consent.

## Results

Responses were received from 73.0% (276/378) Tambons and 59.0% (441/748) staff. The response rate of the THPHs and SAOs were 68.3% (317/464) and 49.3% (140/284), respectively. Forty-one percent (113/275) Tambons included data from both THPH and SAO whereas the rest was reflected solely by THPH viewpoint.

In the focus group discussion, the participants spent almost one hour skimming through the returned questionnaires. Their initial attempt at grouping the informal relationship patterns was a simple count of relationship lines and came up with three categories (Table 1). Their rationale was that each line reflected ‘communication’ or ‘interaction’ between staff of both organizations. They arbitrarily categorized the responses into three levels and rearranged the questionnaires into respective groups.

**Table 1:** **Approach for informal relationship categorization**

**Round**	**# Category**	**Level**	**Definition**	**Frequency (%)**
**1**	3	Strong	>5 relationship lines	113 (39.8)
		Moderate	3-5 relationship lines	88 (31.0)
		Weak	<3 relationship lines	83 (29.2)
**2**	4	Strong	At least 1 relationship line between Chief Executive of SAO and any position of THPH	166 (58.5)
		Moderate	At least 1 relationship line between Deputy Chief Executive of SAO or Chairman of SAO Council and any position of THPH	34 (12.0)
		Weak	Any other relationship line between staff of both organizations	21 (7.4)
		None	No relationship line was drawn	63 (22.2)

The discussion went on with more counter arguments against the applied categorization strategy. Half of the participants thought that each relationship line should not have similar weight. They then discussed alternative approaches based on their experiences. After another hour of discussion, they concluded that the most influential position was the Chief Executive of SAO because he/she had absolute control over administration and budget for the whole Tambon. The Deputy Chief Executive of SAO and Chairman of SAO Council were other important positions that could influence the decision, budget, and management of health services in a Tambon. They then decided to revise the categorization strategy as follows. A strong informal relationship exists when the Chief Executive of SAO has any relationship degree with any THPH staff. When the Deputy Chief Executive of SAO or Chairman of the SAO Council has such a relationship, the Tambon was classified as moderate informal relationship. A Tambon with some other relationship patterns between the staff of both organizations was categorized as a weak informal relationship. With the proposed classifications, approximately 59.1, 21.2, 4.9 and 14.7% of the surveyed Tambon have strong, moderate, weak, and no informal relationship, respectively. At the end of the session, the participants expressed their appreciation of the session as they felt this was the first time they saw harmonized effort to improve Tambon health system.

## Discussion

The findings from this exploratory study suggested that informal relationship between local government agencies do exist and could affect the development and implementation of local health services, especially in the decentralization context. Previous attempts to solve the hurdles of decentralization in Thailand and elsewhere have focused only on formal exchanges and interactions between organizations but have ignored potential informal relationships. The situation has been complicated by unclear roles and responsibilities of local government. The proposed categorization of Tambons was regarded by policymakers as the best available strategy to move health decentralization forward starting with the Tambon with strong informal relationships.

THPH is staffed by health professionals, local non-health executive were believed to have much more influence on the development and implementation of health services as reflected in the discussion among local experts. It can be inferred that, local health service provision will gradually be dominated by management rather than professional perspectives.

This study stressed the importance of inter-organizational relations as a key factor for local health service provision, especially in conjunction with locally influential leaders. Leadership studies are prescriptive in flavor and have concentrated on inter-organizational matters, particularly the psychological elements of a good leader [9]. As local executives are constantly involved in activities that cross the formal boundaries of the organization [2], he or she must establish the terms of exchange with others whose cooperation is important for achieving goals [11]. Also, health service organizations are highly dependent on their institutional environments for legitimacy and funding and they must be able to foresee technological and policy developments that may affect the availability of financial and personnel resources [12]. These external exchanges require the leader to deal with individuals and organizations that are not under his or her direct control.

The impact of informal relationship might be different across contexts. In the Thai culture where people are usually more concerned with familiarity and hospitality than empirical evidence when making decisions, it is not surprising to see a number of large projects that were started from informal connection. While it can help maximize efficiency, informal networks can also cause undesirable consequences as well [10]. This is, however, generally not the case except in rare circumstances. For example, a study of the voting culture of highland people was conducted and found that most samples voted for the lowland candidate because of his or her ability to act as local representative to coordinate with government agencies and other areas [13].

A simple yet innovative methodology was added to the planned national survey of local health systems and could be applied in other contexts. A number of more robust techniques have been used to explore informal relations in the business sector. For example, in order to understand informal connections among company staff, a systematic assessment of advice and trust networks was proposed [10]. However, the method was not feasible for inter-organizational settings. The method used in this study was designed based on the balance between the breadth and the depth of better understanding of informal relationship between local health and non-health organizations. With the proposed categorization, further study could be conducted to assess the association between informal relationship and health outcomes.

## Conclusion

Informal relationships between the staff of local health and non-health agencies can potentially affect the operations and development of the local health services system.

## Additional file

Additional file 1:
**Questionnaire.**
