# Supplementary material for: Informal relationship patterns among staff of local health and non-health organizations in Thailand
Source: BMC Health Serv Res. 2015 Mar 20;15:113. doi: 10.1186/s12913-015-0781-8 (PMC4369356; doi:10.1186/s12913-015-0781-8)
Supplement: Additional file 1: — Questionnaire. [file 12913_2015_781_MOESM1_ESM.docx]

**QUESTIONNAIRE**

**Informal relationship patterns among staff position of local health and non-health organizations in Thailand**

Please draw all possible ‘relationship lines’ between each pair of SAO and THPH staff positions, along with a number on each line that reflect ‘degree of relationship’ (1, friend; 2, second-degree relative; 3, first-degree relative; 4, spouse).

| SAO position | THPH position |
| --- | --- |
| Chief Executive O | O Director |
| Deputy Chief Executive I O | O Registered Nurse |
| Deputy Chief Executive II O | O Technical Public Health Officer |
| Chairman of the Council O | O Community Public Health Officer |
| Vice Chairman of the Council O | O Dental Public Health Officer |
| Chief Administrator O | O Employee |
| Deputy Chief Administrator O | O Worker |
| Chief of Secretary Office O | O others |
| Chief of Finance Division O | O others |
| Chief of Public Work Division O | O others |
| Chief of Public Health Division O | O others |
| Chief of Public Education Division O | O others |
| Council Member O |  |
| Policy and Planning Analyst O |  |
| Community Developer O |  |
| Computer System Officer O |  |
| Disaster Prevention and Alleviation Officer O |  |
| General Service Officer O |  |
| Employee O |  |
| Worker O |  |
| others O |  |

Note: The English terms were officially translated as in government documents. Some incorrect terms were slightly modified to comprehensible for readers.
